# DNA Content Variation and Its Significance in the Evolution of the Genus *Micrasterias* (Desmidiales, Streptophyta)

**DOI:** 10.1371/journal.pone.0086247

**Published:** 2014-01-21

**Authors:** Aloisie Poulíèková, Petra Mazalová, Radim J. Vašut, Petra Šarhanová, Jiøí Neustupa, Pavel Škaloud

**Affiliations:** 1 Department of Botany, Faculty of Science, Palacký University in Olomouc, Olomouc, Czech Republic; 2 Department of Botany, Charles University in Prague, Prague, Czech Republic; 3 Department of Biology, Faculty of Science, University of Hradec Králové, Hradec Králové, Czech Republic; Leibniz-Institute of Plant Genetics and Crop Plant Research (IPK), Germany

## Abstract

It is now clear that whole genome duplications have occurred in all eukaryotic evolutionary lineages, and that the vast majority of flowering plants have experienced polyploidisation in their evolutionary history. However, study of genome size variation in microalgae lags behind that of higher plants and seaweeds. In this study, we have addressed the question whether microalgal phylogeny is associated with DNA content variation in order to evaluate the evolutionary significance of polyploidy in the model genus *Micrasterias*. We applied flow-cytometric techniques of DNA quantification to microalgae and mapped the estimated DNA content along the phylogenetic tree. Correlations between DNA content and cell morphometric parameters were also tested using geometric morphometrics. In total, DNA content was successfully determined for 34 strains of the genus *Micrasterias.* The estimated absolute 2C nuclear DNA amount ranged from 2.1 to 64.7 pg; intraspecific variation being 17.4–30.7 pg in *M. truncata* and 32.0–64.7 pg in *M. rotata*. There were significant differences between DNA contents of related species. We found strong correlation between the absolute nuclear DNA content and chromosome numbers and significant positive correlation between the DNA content and both cell size and number of terminal lobes. Moreover, the results showed the importance of cell/life cycle studies for interpretation of DNA content measurements in microalgae.

## Introduction

Streptophycean green algae are a sister group to land plants [1]–[3]. Desmids are the unicellular representatives of Zygnematophyceae (Streptophyta), and characterized by thousands of morphotypes. They are a logical target group for investigating nuclear DNA content variation [4]. They possess both asexual and sexual reproduction within their life cycle, however sexual reproduction is rare and its study lags behind that of other groups of organisms. Particularly the nuclei behaviour during the reproduction has not been studied in the majority of genera. Thus we still presuppose that desmids with other members of the Zygnematophyceae are haploid in the vegetative stage, only the zygospore being diploid [5]. Although some cases of higher ploidy level in vegetative stage has already been published in the species *Closterium ehrenbergii* [6], the life cycle of this species seems to be extraordinary. In contrast to *C. ehrenbergii* producing 2 zygotes per pairing cells, other genera (*Micrasterias, Cosmarium, Euastrum*) are producing 1 zygote per pair of sexualized cells [7], [8].

Cytogenetic research on Desmids is intriguing due to the presence of holocentric (holokinetic) chromosomes [9] which have a kinetochore located along the whole chromosome. In general, this type of chromosome is uncommon though described in some higher plant families (*Cyperaceae* and *Juncaceae*) and in the genera *Drosera*, *Myristica*, *Cuscuta* and *Chionographis* [10]. It is also described in some algae, arthropods and nematodes, including the model *Caenorhabditis elegans*. By their nature, holocentric chromosomes have high potential for rapid chromosome rearrangements as chromosome fragments can undergo both normal meiosis and mitosis. For this reason, holocentric organisms are characterised by enormous diversity in both number and size of chromosomes. Chromosome number in desmids ranges from 14 to 592 [11] and numbers characteristic of an aneuploid series have also been found [12], [13]. These phenomena suggest that symploidy or agmatoploidy prevails over polyploidy in these taxonomic groups.

Polyploidization, i.e. whole genome duplication (WGD), is considered one of a major evolutionary process in higher plants [14], [15]. Since early reviews of polyploidy in the late 1930's, the number of known polyploid events in the evolution of higher plants has been continuously growing along with the development of new research methods [15]. Recently, with an advent of new sequence techniques it has become evident that by far the vast majority of flowering plants have experienced (ancient) polyploidisation in their evolutionary history [16]–[19]. Polyploid series have their origin in reproductive isolation at different ploidy levels due to unbalanced meiosis. These are often treated as different taxa in higher plants at ranks of varieties to species. Several processes at the genome level, such as the reciprocal loss of duplicated genes (fractionation), gene silencing, chromosome rearrangements and others, might lead to cytotype differentiation at different levels, i.e. morphologically, ecologically. Desmidiales has been characterized by extensive polyploidy, with both inter- and intraspecific variation in chromosome complements reported [20]. Although there is practically no information about natural populations, changes in the chromosome complements of desmid cells have been artificially induced in cultures. Stable polyploid forms (triploid, tetraploid) of numerous *Micrasterias* species have been produced by Waris and Kallio [21]. Diploid cells were always larger than haploid in all *Micrasterias* species in which these conditions were induced [5].

In higher plants, detection of polyploid taxa within polyploid series became a routine procedure with the development of DNA flow-cytometry (FC) using several different protocols [22]–[26]. However, the microscopic size and highly variable cell wall composition of microalgae has limited its use for microalgae [27]–[29]. Thus, study of genome size variation in microalgae lags behind that of higher plants and seaweeds [4], [30]. The multi-step protocol proposed by Mazalová et al. [31] has been found useful for quantification of DNA content in Streptophyta, particularly desmids and a microalgal standard for FC measurement has been suggested.

Recent taxonomic research on microalgae suggests that traditional species/genera boundaries based largely on cell morphology have underestimated the real species diversity [32], [33]. In addition, numerous traditional genera and higher taxa proved to be polyphyletic so that they have to be revised using molecular phylogenetic methods [34]–[36]. *Micrasterias*, a model genus of desmids (Desmidiales, Streptophyta), includes about 60 morphospecies and its cryptic diversity, phylogeny and biogeography have recently been reported [37]–[41]. The results of the recent phylogenetic studies indicate monophyletic origin of the genus followed by substantial morphological transformation of individual infrageneric lineages [36], [37], [40], [42], [43]. According to a multigenic phylogeny of 41 *Micrasterias* taxa, the genus comprises at least eight lineages [40]. Mapping morphological diversification of the genus, on the phylogenetic tree has revealed profound differences in the phylogenetic signal of selected phenotypic features. Whereas the branching pattern of the cells clearly correlates with the phylogeny, the morphological complexity possibly reflects their adaptive morphological response to environmental conditions [40].

**Figure 1:**
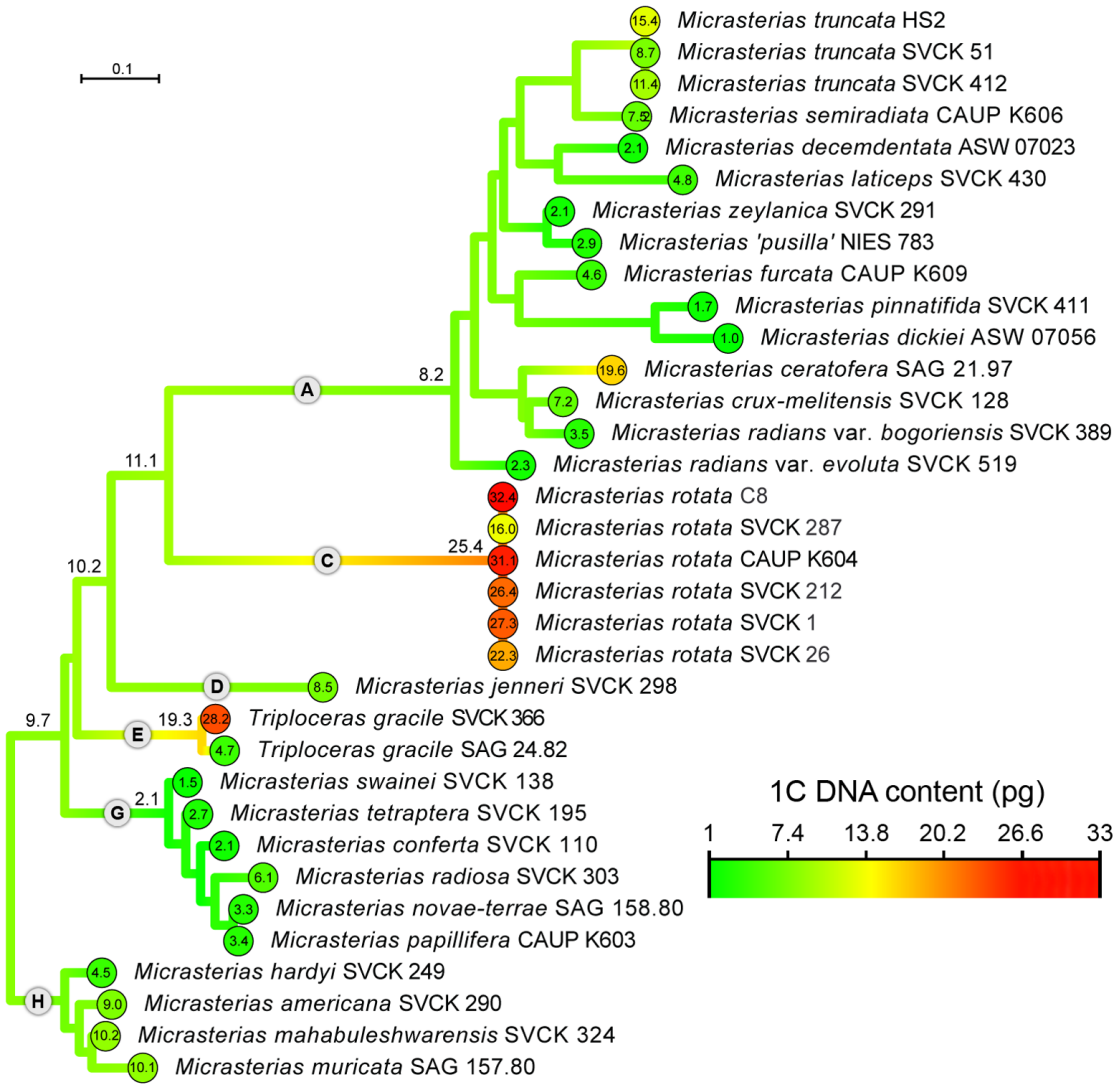
Estimated evolution of DNA content mapped onto the phylogenetic tree of *Micrasterias* (maximum likelihood method). The phylogenetic analysis was conducted on the alignment published by Škaloud et al. [40]. Species affiliation to eight clades (A–H) is indicated. Estimated 1C DNA content is shown at the base of each clade. Scale bar – estimated number of substitutions per site.

Kasprik [44] recognized four groups within the *Micrasterias* species based on chromosome morphology. The first group have small chromosomes with a tendency to aggregation and includes mostly representatives of clade A (, Figure 1), with the exception of *M. americana* from clade H. The second group possessing well-separated chromosomes, includes representatives of clade G (, Figure 1), with the exception of *M. rotata* from clade C. The third group with short, thick, relatively compact chromosomes belongs mostly to clades C and D except for *M. muricata* from clade H (, Figure 1). The fourth group, characterized by long, compact chromosomes which appear to be joined together, includes *M. thomasiana*.

In this study, we asked whether the phylogeny of the genus *Micrasterias* is associated with DNA content variation. To answer this question, we focussed on: 1) assessment of overall DNA content variation; 2) recognition of the significance of DNA content in the evolution at generic and species levels, and 3) the correlations between DNA content and selected cell morphometric parameters.

## Materials and Methods

No specific permits were required for the described field studies. No specific permission was required for any locations and activity. The locations are not privately owned or protected in any way. No activity during field study involved any endangered species or protected species.

### Origin and cultivation of strains

The strains we used were obtained from five public culture collections: Sammlung von Conjugaten-Kulturen, University of Hamburg (SVCK); Culture Collection of Algae, Charles University in Prague (CAUP); Culture Collection of Algae, University of Vienna (ASW), currently deposited in the Culture Collection of Algae at the University of Cologne (CCAC); Culture Collection of Algae, University of Göttingen (SAG); and Microbial Culture collection, National Institute for Environmental Studies, Tsukuba (NIES). Some strains come from the personal collection of Jiří Neustupa [39] (Table 1). They were grown in 50 mm plastic Petri dishes in a liquid oligotrophic medium used in the CAUP culture collection (OGM; [45]). Storage cultures were kept at a temperature of 16°C, under an illumination of 20 ìmol. m^−2^. s^−1^ with 12∶12 light:dark cycle (cooling box Helkama C5G). Subsequently, two weeks before planned flow cytometric measurements, a rich inoculum of each strain (ca 1 ml) was transferred to fresh medium in a 100 mm Petri dishes and kept at a higher irradiation (40 ìmol. m^−2^. s^−1^) with 16∶8 light:dark cycle. All cultures were regularly examined under inverted microscope (Zeiss Axiovert), to check their fitness, cell density and estimation of cell division intensity. Well grown cultures were used for cytometric measurements in exponential phase of growth (14–18 days after subculturing), few slow growing cultures were maintained longer to obtain sufficient cell density. Although the cell density was priority, intensively dividing cultures were preferred because their cell walls were more sensitive to enzymatic disintegration [31]. For three independent repetitions of DNA measurements of each algal strain, we used three Petri dishes of material.

**Table 1:** List of the strains used in this study with GenBank accession numbers.

Species	Strain number	Location	GenBank accession numbers		
			SSU rDNA	psaA	coxIII
*Micrasterias americana*	SVCK 290	Burnham's Swamp at Falmouth, Massachusetts, USA	FR852595	FR852626	FR852666
*M. ceratofera*	SAG 21.97	Sumatra, Indonesia	FR852598	FR852630	FR852670
*M. conferta*	SVCK 110	Moore bei Korvanen, Finland	FR852600	FR852632	FR852672
*M. crux-melitensis*	SVCK 128	A pond in Rheinland, Germany	= AM419206	= FR852633	= FR852673
*M. decemdentata*	ASW 07023	n.a.	FR852602	FR852635	FR852675
*M. dickiei*	ASW 07056	n.a.	FR852623	FR852661	FR852701
*M. furcata*	CAUP K609	Oorid Lough, Connemara, Ireland	FR852605	FR852639	FR852679
*M. hardyi*	SVCK 249	Lake Sorell, Tasmania, Australia	FR852606	FR852640	FR852680
*M. jenneri*	SVCK 298	Mt.Wellington, Tasmania, Australia	FR852607	FR852641	FR852681
*M. laticeps*	SVCK 430	N. Deming Pond, Itasca State Park, Minnesota, USA	FR852608	FR852642	FR852682
*M. mahabuleshwarensis*	SVCK 324	Nacogdoches, Texas, USA	FR852609	FR852643	FR852683
*M. muricata*	SAG 157.80	Texas, USA	FR852610	FR852644	FR852684
*M. novae-terrae*	SAG 158.80	n.a.	FR852611	FR852645	FR852685
*M. papillifera*	CAUP K603	Borkovická Blata, Czech Republic	AM419208	FR852646	FR852686
*M. pinnatifida*	SVCK 411	Laguna de Mucubaj¡Paramo de Mucubaj¡, Merida, Venezuela	FR852612	FR852647	FR852687
*M.* ‘*pusilla*’	NIES 783	Sydney, Centenial Park Australia	= FR852621	= FR852658	= FR852698
*M. radians* var. *bogoriensis*	SVCK 389	Kuching, Borneo, Malaysia	FR852613	FR852648	FR852688
*M. radians* var. *evoluta*	SVCK 519	Lake Ol Bolossat, Kenya	= FR852614	= FR852649	= FR852689
*M. radiosa*	SVCK 303	Lake along the road east of Clifden, Ireland	FR852615	FR852650	FR852690
*M. rotata*	SVCK 287	Burnham's Swamp at Falmouth, Massachusetts, USA	= AM419209	= FR852651	= FR852691
*M. rotata*	SVCK 26	Wildes moor Schwabstedt by Husum, Germany	= AM419209	= FR852651	= FR852691
*M. rotata*	SVCK 212	Timmer Moor near Hamburg, Germany	= AM419209	= FR852651	= FR852691
*M. rotata*	SVCK 1	An unknown locality near Potsdam, Germany	= AM419209	= FR852651	= FR852691
*M. rotata*	C8	A mountain fen near Nové Hamry, Czech Republic	= AM419209	= FR852651	= FR852691
*M. rotata*	CAUP K604	Benthos of flooded quarry pools near Cep village, Czech Republic	AM419209	FR852651	FR852691
*M. semiradiata*	CAUP K606	Peat bog pool in “Borkovická Blata” Nature Reserve, Czech Republic	AM419211	FR852659	FR852699
*M. swainei*	SVCK 138	Summerfield Pond north of Woods Hole, Massachusetts, USA	FR852616	FR852652	FR852692
*M. tetraptera*	SVCK 195	Hickson's Bog I north of Woods Hole, Massachusetts, USA	FR852617	FR852653	FR852693
*M. truncate*	HS2	Pools by Hostens, Aquitaine, France	= FR852620	= FR852657	= FR852697
*M. truncata*	SVCK 51	Zeller Loch by Fulda, Germany	= FR852620	= FR852657	= FR852697
*M. truncata* var. *neodamensis*	SVCK 412	Laguna de Mucubaj¡Paramo de Mucubaj¡, Merida, Venezuela	FR852620	FR852657	FR852697
*M. zeylanica*	SVCK 291	Victoria or New South Wales (Australia)	FR852599	FR852631	FR852671
*Triploceras gracile*	SAG 24.82	Rotary Pond at Falmouth, Massachusetts, USA	AJ428089	EF371259	EF371151
*Triploceras gracile*	SVCK 366	Sumatra, Indonesia	FR852624	FR852662	FR852702

Sources of the strains: (ASW) Algensammlung Wien, University of Vienna, nowadays deposit in the Culture Collection of Algae at the University of Cologne (CCAC); (CAUP) Culture Collection of Algae of Charles University in Prague; (NIES) Microbial Culture Collection, National Institute for Environmental Studies, Japan; (SAG) Sammlung von Algenkulturen Göttingen, Germany; (SVCK) Sammlung von Conjugaten-Kulturen (http://www.biologie.uni-hamburg.de/b-online/d44_1/44_1.htm). The strains not held by above mentioned collections are keeping in personal culture collection of Jiøí Neustupa, Charles University in Prague (Czech Republic).

**= ** identical access numbers with Nemjová et al. [39] and Neustupa et al. [38] – see methods.

### Flow cytometry

Protoplasts were isolated using an enzymatic mixture of 2% Cellulase Onozuka R–10 (Duchefa Biochemie, Netherlands), 0.5% Macerozyme R–10 (Duchefa Biochemie, Netherlands) dissolved in modified rinsing solution PGly [31]. The absolute quantity of nuclear DNA of the algae was estimated by FC using a ML CyFlow instrument (Partec GmbH, Münster, Germany). *Raphanus sativus* cv. Saxa (2C  = 1.11 pg, [46]), *Lycopersicon esculentum* cv. Stupicke (2C  = 1.96 pg, [46]), *Zea mays*, CE–777 (2C  = 5.47 pg, [22]) or *Pisum sativum* cv. Ctirad (2C  = 8.76 pg, [47]) were used as standards. Algal suspension was measured alone at first (without plant standard) to find out the best standard. Young leaves of standard plants were grown separately from algae. They were not affected by enzyme mixture, but chopped with a razor blade in lysis buffer. Then the algal suspension was added to the chopped standard, filtered, incubated and stained. As a fluorescent dye for DNA staining for FC measurements, propidium iodide was used. The whole multi-step protocol has been published in detail elsewhere [31].

**Figure 2:**
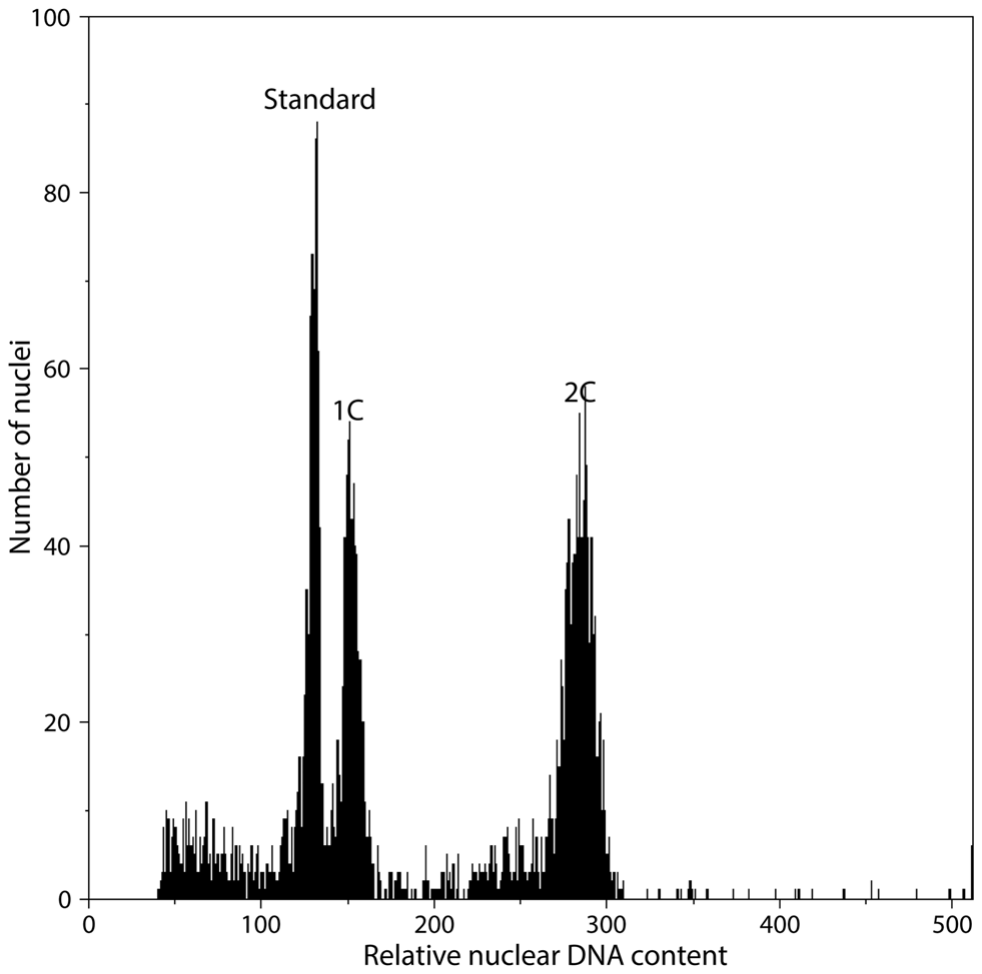
Histogram of relative nuclear DNA content – example. Example of two peaks pattern: strain *M. muricata* SAG 157.80; standard *Pisum sativum*.

All desmid strains were measured at least twice, usually three times, except of strain CAUP K604 with unsufficient amount of desmid cells (extremely slow growth). Altogether 3000 particles were measured (algae + standard together). Each of the repetitive measurements of the same strain was independent (desmid suspension originated from different culture vessels, flow-cytometric measurements were done other day after new calibration of cytometer). Thus the possibilities of errors in DNA measurements were minimized. As the majority of cultures represented proliferating cultures with sufficient amount of dividing cells (synchronized by photoperiod), the histogram of relative nuclear DNA content usually shows 2 peaks – first peak represents vegetative haploid cells (1C), second peak (2C) represent cells in G2 mitosis phase ([48], [49]; Figure 2). The presumption, that the representatives of the genus *Micrasterias* are haploid in vegetative stage is based on the fact, that all published and observed *Micrasterias* species are producing 1 zygote per two pairing cells [7], [8], [50]–[52], no records of two zygotes as in the case of *Closterium ehrenbergii* [6] has been found. All clonal cultures used for measurement were strictly vegetative, neither sexual reproduction nor zygotes were observed during regular microscopic inspection, thus none of the peak represent generative polyploidy caused by hybridization. In few cases the histogram shows 1 peak only. In these cases we used microscopic inspection for decision, whether this peak represents extremely synchronized cells in G2 mitosis phase, or extremely slowly growing strains with dominating vegetative (haploid) cells.

### Phylogenetic analysis, character evolution

The phylogenetic tree used to map the character of the evolution was created using Bayesian inference (BI), MrBayes, version 3.1 [53]. The analysis was conducted on the concatenated SSU rDNA, psaA, and coxIII alignment published by Škaloud et al. [40], reduced to 26 taxa for which the cytometric data were obtained. The genetic identity of *M. ‘pusilla’* (NIES 783) and *M. radians* var. *evoluta* (SVCK 519) strains with those molecularly characterized by Škaloud et al. [40] has been described by Neustupa et al. [37] and Nemjová et al. [39]. Subsequently, eight *M. rotata* and *M. truncata* strains having identical SSU rDNA sequences with those included in the alignment ([38]; [39]) were added to the dataset. The final alignment included 34 taxa.

The existence of a phylogenetic signal in DNA content variation was tested by calculating Pagel's lambda [54]. The maximum likelihood optimization of Lambda value was performed using the ‘phylosig’ function of the phytools package [55]. The ancestral states of DNA content data were calculated using the Ape and Geiger packages [56], [57]. The ancestral states were reconstructed by the function “ace”, using the maximum likelihood optimization. All calculations were done in the program R, ver. 3.0.2. released on 2013-09-25 (website The R Project for Statistic Computing. Available: http://www.r-project.org/. Accessed 2013 Dec 19). The output from R was mapped onto the Bayesian phylogenetic tree with TreeExtender v1.03 [58], using a simple list parser (option – p list). Finally, the evolution of DNA content was traced on a Bayesian phylogenetic tree as colours along a gradient with TreeGradients v1.03 [58].

### Correlation analyses, geometric morphometrics

Correlation analyses were conducted separately on 1) a dataset of all taxa for which the cytometric data were obtained, and 2) a subset of five *M. rotata* strains. The relationship between DNA content and selected cell morphometric parameters (cell length, cell complexity and number of terminal lobes) was evaluated by linear correlation analyses in PAST, ver. 2.01 [59]. The morphometric parameters tested were obtained from Škaloud et al. [40]. To analyze the correlation between the DNA content and cell size in the *M. rotata* subset precisely, we determined the centroid size (CS) of the cells per each strain by a landmark-based shape analysis.

Centroid size, a widely used dimension-free size measure is linearly correlated with traditional univariate cell size measures of desmids, such as cell length or width [60]. It is defined as the square root of the sum of squared distances from all the analyzed landmarks to their centroid [61].

For each *M. rotata* strain, 46–51 randomly chosen cells were photographed. In total, we defined 40 landmarks spanning the outline shape of the cells (see Neustupa et al. [38] and [60]) in TpsDig, ver. 2.16, and the centroid size values were acquired from the general Procrustes analysis of the entire dataset in TpsRelw, ver. 1.49 (website Morphometrics at SUNY Stony Brook. Available: http://life.bio.sunysb.edu/morph/. Accessed 2013 Dec 19.).

## Results

### Overall DNA content variation

In total, DNA content was successfully determined for 34 strains of *Micrasterias* species, including the phylogenetically nested species *Triploceras gracile* (Table 2). To analyse both interspecific and intraspecific DNA content variation, several different strains belonging to the species *M. rotata* and *M. truncata* were analysed, as well. The estimated absolute 2C nuclear DNA quantity varied from 2.1 to 64.7 pg. The smallest genomes belonged to morphologically distinct species *M. dickiei*, *M. swainei*, *M. pinnatifida*, *M. decemdentata* and *M. conferta*. In contrast, the biggest genomes were detected in *M. ceratofera*, one strain of *Triploceras gracile* and several strains of *M. rotata* (Table 2). Generally, the majority of investigated strains possessed rather small DNA content. In fact, the estimated 2C DNA content did not exceed 10 pg in almost half of the strains. Interestingly, high variability in DNA content was also detected among different strains belonging to a single species. In *M. truncata*, the estimated absolute 2C nuclear DNA quantity varied from 17.4 to 30.7 pg; in *M. rotata* strains from 32.0 to 64.7 pg.

**Table 2:** DNA content measurements in *Micrasterias* strains.

Species	Strain number	Peak†	Measurement			Average	STD	Standard species
			1	2	3			
*Micrasterias americana*	SVCK 290	G2/2	9.04	9.22	8.82	9.03	0.16	*Z. m.*
		G2	18.09	18.43	17.64	18.05	0.33	
*M. ceratofera*	SAG 21.97	G2/2	19.60	19.70	19.55	19.62	0.06	*P. s.*
		G2	39.19	39.40	39.10	39.23	0.13	
*M. conferta*	SVCK 110	G2/2	2.06	2.07	2.06	2.06	0.01	*Z. m.*
		G2	4.11	4.15	4.11	4.12	0.02	
*M. crux-melitensis*	SVCK 128	G1	7.21	7.20	7.07	7.16	0.07	*L. e.*. *Z. m.*
		G1*2	14.42	14.39	14.13	14.32	0.13	
*M. decemdentata*	ASW 07023	G1	2.03	2.08	2.02	2.05	0.03	*P. s.*. *Z. m.*
		G2	4.09	4.10	4.10	4.09	0.01	
*M. dickiei*	ASW 07056	G2/2	1.02	1.02	1.03	1.02	<0.01	*P. s.*
		G2	2.04	2.05	2.05	2.05	<0.01	
*M. furcata*	CAUP K609	G2/2	4.68	4.45	4.55	4.56	0.09	*L. e.*. *Z. m.*
		G2	9.35	8.90	9.10	9.12	0.19	
*M. hardyi*	SVCK 249	G1	4.57	4.45	4.53	4.52	0.05	*P. s.*
		G1*2	9.15	8.91	9.06	9.04	0.10	
*M. jenneri*	SVCK 298	G1	8.34	8.71	8.46	8.50	0.15	*Z. m.*
		G1*2	16.68	17.42	16.92	17.01	0.31	
*M. laticeps*	SVCK 430	G2/2	4.75	4.71	4.93	4.80	0.10	*L. e.*. *P. s.*
		G2	9.50	9.42	9.86	9.59	0.19	
*M. mahabuleshwarensis*	SVCK 324	G1	10.19	10.30	10.16	10.21	0.06	*Z. m.*
		G2	20.38	20.59	20.31	20.43	0.12	
*M. muricata*	SAG 157.80	G2/2	10.50	9.56	10.20	10.09	0.39	*Z. m.. P.s.*
		G2	21.01	19.12	20.41	20.18	0.79	
*M. novae-terrae*	SAG 158.80	G1	3.21	3.42	3.16	3.26	0.11	*P. s.*. *Z. m.*
		G1*2	6.42	6.84	6.32	6.53	0.23	
*M. papillifera*	CAUP K603	G1	3.12	3.12	4.08	3.44	0.45	*P. s.*. *Z. m.*
		G1*2	6.25	6.24	8.15	6.88	0.90	
*M. pinnatifida*	SVCK 411	G1	1.75	1.72	1.71	1.72	0.02	*L. e.. P. s.. R. s.. Z. m.*
		G2	3.39	3.36	3.43	3.40	0.03	
*M. ‘pusilla’*	NIES 783	G1	2.83	3.04		2.94	0.11	*Z. m.*
		G2	5.71	5.65	5.88	5.75	0.10	
*M. radians* var. *bogoriensis*	SVCK 389	G1		3.50	3.42	3.46	0.04	*Z. m.*
		G2	6.34	6.80	6.66	6.60	0.19	
*M. radians* var. *Evoluta*	SVCK 519	G1	2.35	2.11	2.30	2.25	0.10	*P. s.*
		G2	4.59	4.23	4.60	4.47	0.17	
*M. radiosa*	SVCK 303	G2/2	6.02	6.07	6.11	6.07	0.03	*Z. m.*
		G2	12.04	12.14	12.21	12.13	0.07	
*M. rotata*	SVCK 287	G1	16.04	15.93	16.09	16.02	0.06	*Z. m.*
		G1*2	32.08	31.86	32.18	32.04	0.13	
*M. rotata*	SVCK 26	G1	20.88	23.83	22.24	22.32	1.21	*Z. m.*
		G1*2	41.75	47.66	44.48	44.63	2.41	
*M. rotata*	SVCK 212	G1	26.49	26.37	26.19	26.35	0.12	*Z. m.*
		G1*2	52.98	52.74	52.38	52.70	0.25	
*M. rotata*	SVCK 1	G1	27.80	27.49	26.66	27.32	0.48	*Z. m.*
		G1*2	55.60	54.97	53.32	54.63	0.96	
*M. rotata*	C8	G1	30.65	33.56	32.88	32.37	1.25	*Z. m.*
		G1*2	61.30	67.12	65.76	64.73	2.49	
*M. rotata*	CAUP K604	G1	31.09			31.09	<0.01	*P. s.*
		G1*2	62.18			62.18	<0.01	
*M. semiradiata*	CAUP K606	G1		7.61	7.36	7.48	0.13	*P. s.*
		G2	14.17	15.14	14.58	14.63	0.40	
*M. swainei*	SVCK 138	G1	1.53		1.53	1.53	<0.01	*Z. m.*
		G2	3.02	3.00	3.19	3.07	0.08	
*M. tetraptera*	SVCK 195	G2/2	2.69	2.74	2.70	2.71	0.02	*P. s.*
		G2	5.38	5.49	5.39	5.42	0.05	
*M. truncata*	HS2	G1	15.34	15.38	15.34	15.35	0.02	*Z. m.*
		G1*2	30.68	30.76	30.68	30.71	0.04	
*M. truncata*	SVCK 51	G2/2	8.93	8.42	8.67	8.68	0.21	*P. s.*
		G2	17.86	16.84	17.35	17.35	0.42	
*M. truncata* var. *neodamensis*	SVCK 412	G1		11.39		11.39	<0.01	*Z. m.*
		G2	21.81	21.73		21.77	0.04	
*M. zeylanica*	SVCK 291	G1		2.07	2.16	2.11	0.04	*L. e.*. *Z. m.*
		G2	4.29	4.17	4.26	4.24	0.05	
*Triploceras gracile*	SAG 24.82	G2/2	4.64	4.72	4.72	4.69	0.04	*P. s.*. *Z. m.*
		G2	9.29	9.44	9.44	9.39	0.07	
*Triploceras gracile*	SVCK 366	G1	28.06	28.35		28.20	0.14	*P. s.*
		G1*2	56.12	56.69		56.41	0.29	

† G1, G2 – measured values, G1*2; G2/2 – counted values from G1, G2; (AVG) average, (STD) standard deviation.

Standards: *L. e.  =  Lycopersicon esculentum, P. s.  =  Pisum sativum, R. s.  =  Raphanus sativus, Z. m.  =  Zea mays*.

**Figure 3:**
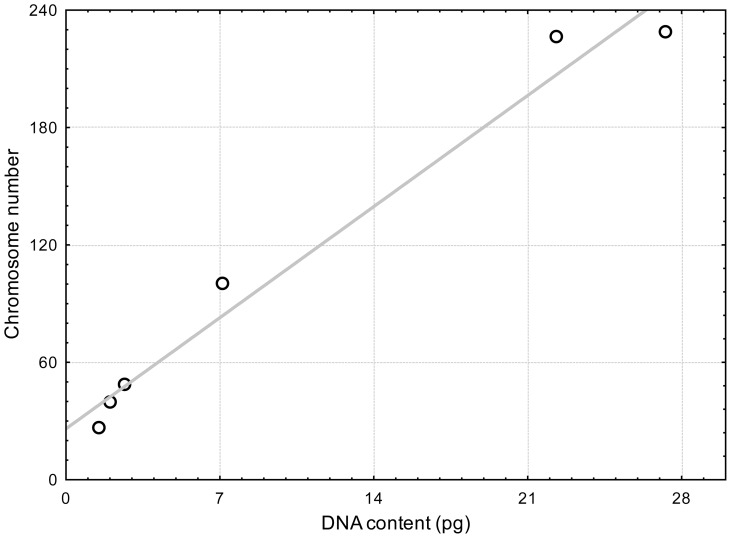
Linear regression of measured DNA content on the chromosome data in six *Micrasterias* strains. Chromosome data published by Kasprik [44], (r = 0.987, R^2^  = 0.974, P-value<0.001).

To test the correlation between the estimated DNA content values and real number of chromosomes, we compared our measured values with the chromosome data published by Kasprik [44]. For the purpose of this test, we used six *Micrasterias* SVCK strains used in both studies (Table 2 and 3). As can be seen from the regression analysis (Figure 3), a linear regression fits the data very well (R^2^ = 0.974, P-value<0.001), suggesting a strong correlation between absolute nuclear DNA content and chromosome number.

**Table 3:** Cytogenetic and morphometric characteristics of the analyzed strains. Species names are given in Table 1.

Strain number	Chromosome number^1^	Average 1C DNA cont. (pg)	Centroid size	Number of terminal lobes per semicell^2^	Cell complexity^2^	Cell length^2^
SVCK 290	-	9.03	-	4	1.59064	140
SAG 21.97	-	19.62	-	2	1.448164	131
SVCK110	39	2.06	-	8	1.623739	90
SVCK 128	∼100	7.16	-	4	1.845687	107
ASW 07023	-	2.05	-	2	1.334061	50
ASW 07056	-	1.02	-	0	0.75505	39
CAUP K609	-	4.56	-	4	2.545707	153
SVCK 249	-	4.52	-	2	2.143069	216
SVCK 298	-	8.50	-	8	1.341453	157
SVCK 430	-	4.80	-	1	1.185784	150
SVCK 324	-	10.21	-	2	1.802018	160
SAG 157.80	-	10.09	-	2	1.58329	175
SAG 158.80	-	3.26	-	6	2.242455	124
CAUP K603	-	3.44	-	8	2.299155	130
SVCK 411	-	1.72	-	1	1.220962	61
NIES 783	-	2.94	-	4	1.348246	60
SVCK 389	-	3.46	-	6	2.355276	102
SVCK 519	-	2.25	-	4	1.999237	107
SVCK 303	-	6.07	-	14	3.135475	175
SVCK 287	-	16.02	3369.2	12	2.164567	260
SVCK 26	226	22.32	3417.4	12	2.164567	260
SVCK 212	-	26.35	-	12	2.164567	260
SVCK 1	229	27.32	3763.7	12	2.164567	260
C8	-	32.37	4341.8	12	2.164567	260
CAUP K604	-	31.09	4421.9	12	2.164567	260
CAUP K606	-	7.48	-	4	1.482767	96
SVCK 138	26	1.53	-	10	3.142072	155
SVCK 195	48	2.71	-	8	2.056462	116
HS2	-	15.35	-	2	1.278057	100
SVCK 51	-	8.68	-	2	1.278057	100
SVCK 412	-	11.39	-	2	1.278057	100
SVCK 291	-	2.11	-	2	1.169259	57
SAG 24.82	-	4.69	-	0	0.6973971	400
SVCK 366	-	28.20	-	0	0.6973971	400

1 according to Kasprik [44]; ^2^ according to Škaloud et al. [40].

### Evolution of DNA content

To better understand the evolutionary history of DNA content changeover during the diversification of the genus *Micrasterias*, we mapped the estimated DNA content values along the phylogenetic tree. A phylogram was constructed by the Bayesian inference method, based on the concatenated SSU rDNA, psaA, and coxIII alignment published by Škaloud et al. [40]. Since the protoplast isolation and subsequent DNA content determination was not successful in all strains [31], our phylogram contains only 26 of 36 *Micrasterias* species with known sequence data. The topology of the resulting phylogenetic tree was highly congruent with the phylogram constructed by Škaloud et al. [40], with strains inferred as members of 6 main clades (A, C, D, E, G, and H).

The existence of a phylogenetic signal in nuclear DNA content was tested using Pagel's lambda calculations. The related species were significantly different in their DNA content (ë = 0.413, p-value = 0.34). However, the existence of a phylogenetic signal was revealed by the maximum likelihood reconstruction of the ancestral states (Figure 1). Whereas all species of clade G showed a clear tendency to rather low DNA content, all six *M. rotata* strains forming the clade C tended to have much higher amounts of DNA. On the other hand, clear differences were found in some closely related strains, indicating potential rapid evolutionary dynamics in the genome size. For example, two *Triploceras gracile* strains differed six times in their 2C nuclear DNA content (9.4 and 56.4 pg, respectively). Similarly, *M. crux-melitensis* (14.3 pg) and *M. radians* var. *bogoriensis* (6.6 pg) had 2C DNA content lower than the closely related *M. ceratofera* (39.2 pg).

### Correlations between DNA content and cell morphometric parameters

**Figure 4:**
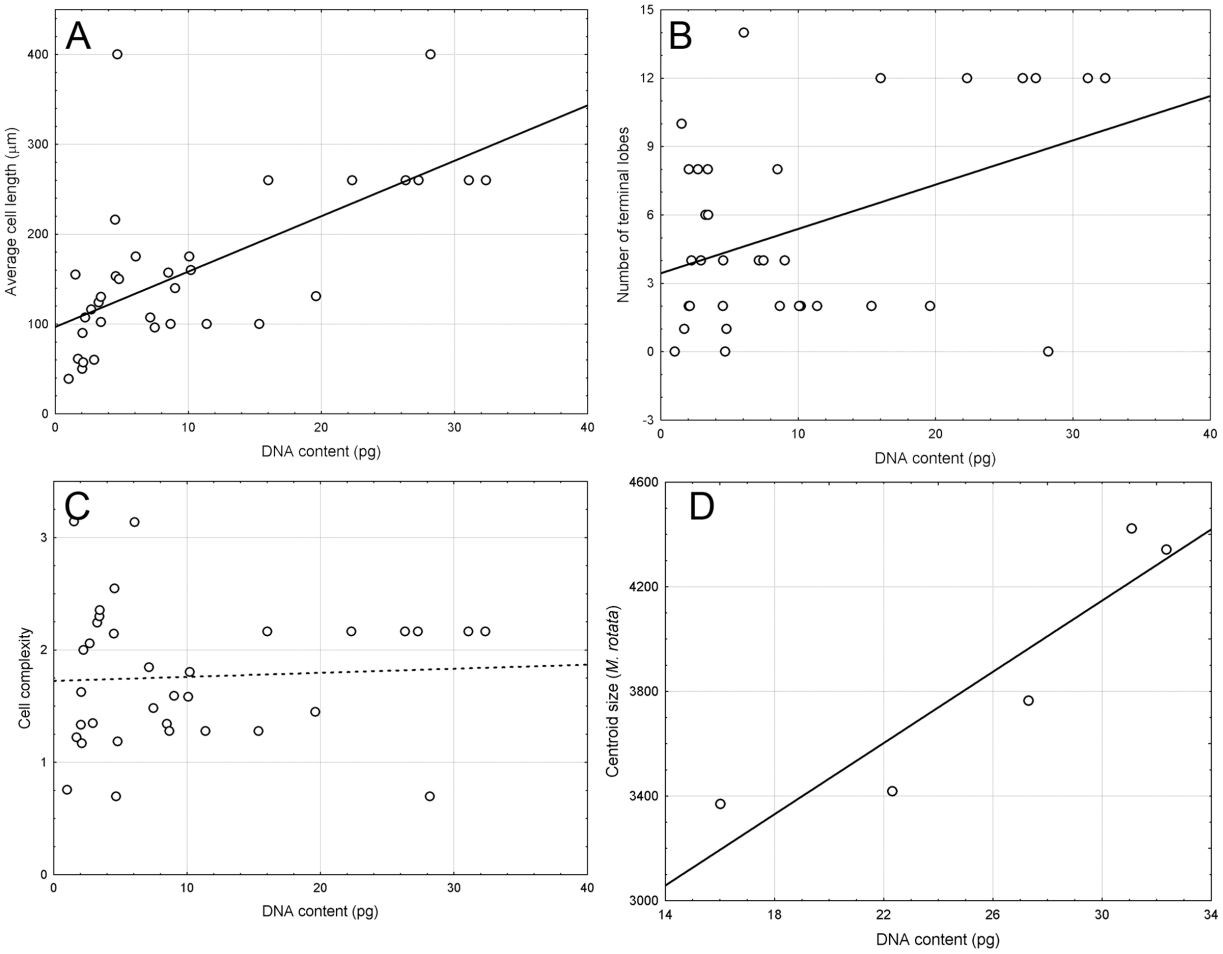
Correlation of 1C DNA content and morphometric parameters. (A) average cell length, (B) number of terminal lobes, (C) cell complexity, (D) centroid size in *Micrasterias* strains. Significant correlations are given by solid lines.

In addition to analysis of the evolutionary significance of the DNA content variation, correlations between the genome size and selected morphometric characteristics were determined. Statistical relationships, described by correlation coefficients between the estimated DNA content and cell length, cell complexity and number of terminal lobes, are presented in Table 4 and illustrated in Figure 4A–C. A significant correlation was found between the DNA content and both average cell length (Figure 4A) and number of terminal lobes (Figure 4B). The correlation with the average cell length was the most significant. In fact, 42.3% of the overall variability in cell lengths could be explained by the genome size data.

**Table 4:** Correlation between the 1C DNA content and selected cell morphometric parameters.

Trait	R	R^2^	p-value
***Micrasterias*** ** dataset**			
Cell length	0.651	0.423	**<0.001**
Cell complexity	0.056	0.003	0.751
Number of terminal lobes	0. 426	0.181	0.012
***Micrasterias rotata*** ** dataset**			
Centroid size	0.919	0.845	**0.027**

Correlation analyses were performed for the whole dataset (34 strains) as well as for the *Micrasterias rotata* subset (6 strains). Number of specimens/cells analysed morphologically for each strain: N = 46–51.

As mentioned above, significant differences in DNA content were also found between different strains of a single species. To test whether these differences could also affect the cell size, we analyzed the correlation between DNA content and centroid size in five *M. rotata* strains having identical SSU rDNA sequences. Strong positive correlation was found (Table 3; Figure 4D) and altogether 84.5% of cell size variability detected could be explained by the different nuclear DNA contents of analyzed strains.

## Discussion

### Overall DNA content variation and correlations with chromosome numbers

Genome size measurement in algae is presently at the beginning and, frequently methodologically more demanding than in embryophytes. This is also reflected in the amount of the Kew Plant DNA C-values database algae entries of only 253, in contrast to the presently over 7500 angiosperm C-value entries [62]. The results reported in this paper constitute one of few flow cytometric analysis in microalgae [31], [63]–[65] and first such analysis in desmids.

Measurements of DNA content in 34 strains of the genus *Micrasterias*, ranging from 2C = 2.1 to 64.7 pg, are in congruence with previously published data, based mostly on microspectrophotometric methods.

For Streptophyta, 2C nuclear DNA content range from 0.2 to 6.4 pg, excluding the highly polyploid Charales and Desmidiales, which have genome sizes of up to 14.8 and 46.8 pg, respectively. In general, nuclear DNA content is smaller in Zygnematales than in Desmidiales [30]. Algae are a highly diverse organism group and phylogenetically at the basis of land plants. Therefore, a broader knowledge of genome size would be enormously valuable for the evaluation of the role of nuclear DNA amount in evolution. Nuclear DNA content data for Streptophyta superimposed on a contemporary molecular phylogeny indicated that early diverging lineages, including some members of Chlorokybales, Coleochaetales and Klebsormidiales, have genomes as small as 2C = 0.1–2.7 pg [30], [31]. It has been proposed that the Streptophyte ancestral nuclear genome, common to both the charophyte and the embryophyte lineages, can be characterized as 1C = 0.2 pg and 1n = 6. Moreover, the DNA contents of the freshwater charophyceans and zygnemataleans are significantly larger than that of Rhodophyta (2C = 0.2–2.8 pg) and Phaeophyta (2C = 0.2–1.8 pg) [4]. Although greater values for DNA content exist in polyploid bryophytes, more than 80% of the nuclear DNA 1C-values in mosses have been reported to occur in a narrow peak between 0.25 and 0.6 pg [66]. The size of algal genomes is best appreciated when compared with the minimum amount of DNA estimated in angiosperms. The smallest angiosperm genome is known in the carnivorous plant species *Genlisea margaretae* Hutch. (Lentibulariacea) with 1C = 0.065 pg equalling ca. 63.4 Mbp [67]. The genome of the genetic model species, *Arabidopsis thaliana* (L.) Heynhold with five chromosomes only, is approximately only 1C = 157 bp large (0.16 pg) [68].

Many of the Zygnemataceae appear to be characterized by polyploid „species complexes“ [20] and the large cell sizes reported for many Desmidiales, suggest that polyploidy in these uninucleate, unicellular organisms has produced some of the largest nuclear genome sizes known in plants. These suggestions are in congruence with our measurements. Broad variation has been found in different clones of the same species: *Micrasterias rotata*, *M. truncata*, and *Triploceras gracile* (Tables 2 and 3). Angiosperms with holocentric chromosomes have in general, large genome size variation. Individual species of the genus *Carex* have almost 8-fold variation in their 4C nuclear DNA content [69], genus *Luzula* up to 6-fold variation in their 2C nuclear DNA content [70], [71]. Likewise, in the genus *Schoenus* up to 14.8-fold variation in 2C DNA content [72] and in *Eleocharis* even up to 22.1-fold (0.25 pg in *E. acicularis* and 5.53 pg in *E. palustris*; [73]) were reported.

Although some of the desmid strains were maintained as long as 40 years and changes in chromosome numbers should be expected in cultures, our results support the earlier findings within Desmidiales [4] that chromosome complements and nuclear DNA contents are highly correlated, providing circumstantial evidence for the pervasive role of polyploidy in the evolution of this group of algae. In contrast, in the marine species of Ulvophyceae there is low correlation, consistent with a high occurrence of aneuploidy, i.e. chromosomal fusion and/or fission events. On the larger scale, genome variation in holocentric genera does not always correlate with chromosome numbers. Although the chromosome number in *Carex* varies greatly, between 6 and 62, genome size remains nearly constant [10], [74]. Similar dissonance between the chromosome number and DNA content is found in another holocentric organisms, such as *Schoenus* [72] and *Juncus* [75].

The dynamic nature of holocentric chromosomes can be demonstrated by the nuclear DNA variation within a single variety of sedge species *Carex scoparia* var. *scoparia*, in which the chromosome counts varies from 2n = 62 to 2n = 68 and the 1C DNA content varies from 0.342 pg to 0.361 pg [76].

Because of overall lack of experience with flow cytometric measurements of the DNA content in microalgae, we were solving a lot of methodological problems, during our study. Beside the problems with nuclei isolation due to the complexity of cell wall discussed previously [31] we noticed the peak pattern variation in histograms obtained from individual measurements.

Histograms of well growing cultures in exponential phase of growth showed two peaks, first representing vegetative cells (G1), second representing dividing cells (G2). We believe, that for desmid genus *Micrasterias* first peak represent haploid cells, i.e. 1C DNA content. The height differences of both peaks (G1>G2 or G1<G2) depend on stage of cell cycle [65]. Although *Chlamydomonas* represent different microalgal group and type of cell/life cycle, authors found that G1 peak is always observable and never represents less than 29%, because in each stage exist some cells, which are not dividing. The percentage of both peaks depend on degree of synchrony and division rate which both depend on complex of factors including culture conditions and cell size [65]. Thus flow cytometric measurements should be accompanied by careful inspection of measured material, which serve as a base for interpretation of such histograms, which show 1 peak only (see Methods).

The exact knowledge of cell cycle and degree of synchrony of each species/sample should lead to exact interpretation, but represent another complication which makes flow cytometry in microalgae extremely time consuming. Moreover, the lack of information on reproduction of the majority of microalgal species, complicate our interpretation of ploidy level. Vegetative cells (G1) can be haploid (some desmids) or diploid (e.g. diatoms), depending on their life histories.

### Significance of DNA content in evolution

Genome size is an important species-specific characteristic in organisms with centromeric chromosomes and was extensively studied in past decade in order to describe its changes during the evolution of angiosperms [77]. The correlation between the C-value and other traits was extensively investigated, not only from the taxonomic point-of-view, but also from a broader biological perspective. In general, the DNA amount serves as a reliable taxonomic indicator which can help to understand the taxonomic problems in angiosperms [78]. Unfortunately limited data are available for microalgae.

Some studies on holocentric angiosperms [72] suggest that some clades demonstrate a narrow range of genome size variability, whereas others exhibit great variation. Although, this phenomenon can be found in taxa with monocentric chromosomes too it seems to be more typical/frequent in holocentric genera. Indeed, similar results were obtained in *Micrasterias*. For example, clade G consists of strains with a narrow range of genome size variability. However, within other clades, clear differences were found in some closely related strains, indicating the potentially rapid evolutionary dynamics in the genome size. Even more, great variability in genome size was detected in different strains of a single species (*Micrasterias rotata*, *M. truncata* and *Triploceras gracile*).

### Influence of DNA content on cell size and morphology

Small genome size in angiosperms appears to be correlated with phenotypic characteristics such as rapid seedling establishment, short minimum generation times, reduced cost of reproduction and increased reproductive rate [79]–[82]. It has been recognized that although nuclear genome size is highly correlated with many cellular and ecological parameters, „correlation“ and „causation“ are far from interchangeable [83]–[85].

Ploidy level in conjugating green algae may be of taxonomic significance as cell dimensions are considered to be diagnostic [20] and highly correlated with genome size [86]. Diploid cells in *Micrasterias* were found to be usually larger than that of haploids. Waris and Kallio [21] and Brandham [87] observed that larger cells of *Closterium, Cosmarium* and *Staurastrum* were polyploid.

Kasprik [44] reported an interesting case of an aneuploid series correlated with morphological differences in *Micrasterias thomasiana*. The basic chromosome number of this desmid is n = 39, but morphologically irregular variants contained n = 40, n = 46 or even n = 70 and 75. Some of the cells of this latter clone were inclined to develop more or less typical morphologies, except that they were significantly larger [44]. Correlations between the DNA content and cell length, cell complexity and number of terminal lobes were tested in this study. The significant positive correlations were found between the DNA content and both average cell length and number of terminal lobes. Changes in the degree of radiation in some desmids, result from an increase in the level of ploidy, as concluded by Starr [88] in his study of a heterothallic strain of *Cosmarium turpinii*. Starr considered the production of large forms to be a response to increase in nuclear quantity; whereas the change in shape (bi-, tri-, quadriradiate cells) he explained as a response to the increase in cell volume.

We can support partially this hypothesis by our data. In contrast to the number of terminal lobes variation, cell complexity was not significantly correlated with DNA content.

## Conclusions

This is the first flow cytometric analysis of both interspecific and intraspecific DNA content variation within one microalgal genus. There was strong correlation between nuclear DNA content and chromosome number in strains of the genus *Micrasterias* and between DNA content and cell size and morphology in the species *Micrasterias rotata*.

Moreover, the results showed the importance of cell/life cycle studies for interpretation of DNA content measurements in microalgae.
