# Correction: DNA Content Variation and Its Significance in the Evolution of the Genus *Micrasterias* (Desmidiales, Streptophyta)

**DOI:** 10.1371/journal.pone.0092399

**Published:** 2014-03-10

**Authors:** The PLOS ONE Staff

The names of the first and fifth authors are spelled incorrectly. The first author's correct name is: Aloisie Poulíčková. The fifth author's correct name is: Jiří Neustupa.

The correct citation is: Poulíčková A, Mazalová P, Vašut RJ, Šarhanová P, Neustupa J, et al. (2014) DNA Content Variation and Its Significance in the Evolution of the Genus *Micrasterias* (Desmidiales, Streptophyta). PLoS ONE 9(1): e86247. doi:10.1371/journal.pone.0086247.

In addition, there are multiple instances of incorrect symbols used in terms in the Materials and Methods.

The fourth and fifth sentences of the "Origin and cultivation of strains" section of the Materials and Methods should read: "Storage cultures were kept at a temperature of 16°C, under an illumination of 20 µmol. m^-2^. s^-1^ with 12:12 light:dark cycle (cooling box Helkama C5G). Subsequently, two weeks before planned flow cytometric measurements, a rich inoculum of each strain (ca 1 ml) was transferred to fresh medium in a 100 mm Petri dishes and kept at a higher irradiation (40 μmol. m^-2^. s^-1^) with 16:8 light:dark cycle."

In addition, there was an incorrect use of a symbol in a term in the Results.

The second sentence of the second paragraph of the "Evolution of DNA content" section of the Results should read:

"The related species were significantly different in their DNA content (λ =  0.413, p-value  = 0.34)."
